# Semi-Automated Field Plot Segmentation From UAS Imagery for Experimental Agriculture

**DOI:** 10.3389/fpls.2020.591886

**Published:** 2020-12-09

**Authors:** Ciaran Robb, Andy Hardy, John H. Doonan, Jason Brook

**Affiliations:** ^1^Earth Observation Lab, Department of Geography and Earth Sciences, Aberystwyth University, Aberystwyth, United Kingdom; ^2^The National Plant Phenomics Centre, Institute of Biological, Environmental and Rural Sciences (IBERS), Aberystwyth University, Aberystwyth, United Kingdom

**Keywords:** crop plot, segmentation, UAS, structure-from-motion, edge-detection, Hough-transform

## Abstract

We present an image processing method for accurately segmenting crop plots from Unmanned Aerial System imagery (UAS). The use of UAS for agricultural monitoring has increased significantly, emerging as a potentially cost effective alternative to manned aerial surveys and field work for remotely assessing crop state. The accurate segmentation of small densely-packed crop plots from UAS imagery over extensive areas is an important component of this monitoring activity in order to assess the state of different varieties and treatment regimes in a timely and cost-effective manner. Despite its importance, a reliable crop plot segmentation approach eludes us, with best efforts being relying on significant manual parameterization. The segmentation method developed uses a combination of edge detection and Hough line detection to establish the boundaries of each plot with pixel/point based metrics calculated for each plot segment. We show that with limited parameterization, segmentation of crop plots consistently over 89% accuracy are possible on different crop types and conditions. This is comparable to results obtained from rice paddies where the plant material in plots is sharply contrasted with the water, and represents a considerable improvement over previous methods for typical dry land crops.

## 1. Introduction

With a growing global population and the impact of global warming, food security is one of the major issues faced globally today, central to which is the improvement of agricultural practices (Valluru et al., 2015). State of the art technology plays a vital role in this improvement, harnessing intelligence derived from sensor systems to inform management and monitoring (Mavridou et al., 2019). This has included the use of Unmanned Aerial Systems (UAS) for capturing data over fields and plots in both commercial and experimental. UAS can provide data and information comparable to field sources but are often cheaper than field-based methods; providing a cost effect means of monitoring crops and predicting yield (Zhang and Kovacs, 2012; Manfreda et al., 2018; Zhao et al., 2019).

UAS-derived imagery typically provides both optical and modeled height information through structure from motion (SfM), both potentially useful indications of crop state. SfM is a process utilizing techniques from computer vision and photogrammetry to reconstruct 3-D scenes from collections of overlapping photos (Rupnik et al., 2017; Schönberger, 2018). Whilst the direct measurement of crop height is available through UAS-borne LiDAR, these are currently limited due to the size and expense of LiDAR instruments. For the moment therefore, UAS-borne optical sensors remain the more widely used, economical solution to deriving spectral and height-based data and the majority of research and industry has used these data types. A common approach by many academic studies and commercial applications is acquire imagery using multi-spectral sensors and derive radiometric indices (such as NDVI) on a per-pixel basis as a proxy indication of vegetation vigor (Lelong et al., 2008; Sankaran et al., 2015; Matese et al., 2017; Manfreda et al., 2018; Zhao et al., 2019). Where a multi-spectral sensor is not available, others rely on similar RGB-based indices or color transformations to the same end (Bai et al., 2013; Hassanein et al., 2018; Wahab et al., 2018). Either-way, such approaches are only viable if the baseline crop conditions are well-enough related to pixel values, which is more likely with well-separated spectral bands.

Crop canopy height information also serves as an indicator of health/vigor and has the advantage of not requiring a spectral baseline calibration (Bendig et al., 2014; Madec et al., 2017; Zhao et al., 2019). However, with SfM-derived data, canopy height must be averaged over discrete area units to provide meaningful information. Therefore, using the geographical unit of the plot itself may be critical to making remote inferences about differences in crop performance (Khan and Miklavcic, 2019). Generalized plot information provides a more tangible link between UAS imagery-derived metrics and information important to growers, such as the average canopy height, density and ultimately above ground biomass. Discrete crop plots are often used in experimental and breeding contexts to assess the performance of 100's to 1000s of crop varieties under different treatment regimes, over extensive areas (Sankaran et al., 2015; Khan and Miklavcic, 2019). Therefore, analysis is highly dependent on a reliable approach for delineating the plots. This can be done manually but is labor intensive and prone to error. The automation of both the segmentation of discrete plots from imagery and extraction of relevant pixel data would therefore serve as a useful tool for experimental regimes.

The mapping and assessment of crops at close-range has been widely studied using vehicles and increasingly, UAS. The segmentation of individual plants using close proximity photography in order to assess condition has been demonstrated effectively by Bai et al. (2013) using a combination of color transformation, morphological filtering and image thresholding. The well-spaced planting patterns and homogeneous water background of the rice crops in question lend themselves well to these techniques achieving segmentation accuracies of 87–90%. At a similar range, more complex methods using machine learning have been applied to plant and weed segmentation with convolutional neural nets (CNN) emerging most recently, producing similar or slightly improved segmentation accuracy at the cost of algorithm training labor (Knoll et al., 2018; Mavridou et al., 2019; Bosilj et al., 2020). The detection of crop planting lines at close range was carried out by Vidović et al. (2016) through a combination of template matching an energy minimization. Indeed, a number of studies use Hough-based approaches to delineate planting lines and are usually aimed at integration into machinery for guidance including (Ji and Qi, 2011; Mavridou et al., 2019).

In the context of UAS survey-derived imagery, Hassanein et al. (2018) develop a semi-automated technique to segment crop rows based on the color transform of RGB imagery and interpretive pixel thresholds. Machine learning based methods are popular for crop segmentation such as Chen et al. (2017) using a Bayesian classifier on emergent cotton and Pérez-Ortiz et al. (2016) using image segmentation by Bunting et al. (2014) followed by Support Vector Machine classification of the resulting segment attributes to map both crop and adjacent weeds. Utilizing multi-spectral imagery, Dyson et al. (2019) segment rows of crops from UAS-imagery using a deep learning approach which utilized a combination of DSM and optical data (specifically NDVI). In both close-range and UAS-borne contexts, machine learning based approaches as in Bosilj et al. (2020), Knoll et al. (2018), Dyson et al. (2019), and Guo et al. (2018) require extensive training sets to be effective—particularly with deep learning-based models and if the intention is to map crops through changing phenology and locations, where spectral and spatial properties will change. Ideally, it would be preferable to avoid such laborious pre-processing and have an algorithm that is generally applicable without resorting to dataset specific training.

Many research and breeding projects require a per plot assessment of different crop treatments, particularly when the crop in question is closely planted making individual plant segmentation impractical from UAS-borne imagery on an image wide basis. Hence, automatic segmentation of the plots has the potential to save time and effort in the field as well as enhance studies such as Guo et al. (2018), who treat plot segmentation as a manual component of the workflow. Recent efforts at segmenting crop plots are still largely manual, necessitating the user to define the pattern, dimensions and size of the plot grid as well as positioning it by hand over the field of interest (Khan and Miklavcic, 2019; Tresch et al., 2019; Matias et al., 2020). Khan and Miklavcic (2019) employ a fixed grid of plots of specified dimensions constructed by the user, to demarcate crop plots and offer a graph-based energy minimization procedure to fine tune their position using underlying spectral indices. This approach requires the user to specify the complete spatial characteristics of the grid and approximate alignment in advance and utilizes proprietary software. Similarly, a recent tool for demarcating crop plots by Matias et al. (2020) provides functionality for largely manual delineation of crop plots. A similar work by Tresch et al. (2019) requires training samples for the initial delineation of vegetation, then the manual designation of the plot columns, rows and orientations. Such approaches would be improved by direct segmentation of the plots without multiple stages of user-construction. The aforementioned approaches rely on the presence of completely discrete crop plots in order to perform segmentation or classification. The ability to demarcate crop plots or rows based on the only the partial presence of plots and soil/furrow pattern would therefore expand on the attempts of previous studies reliant on the presence of vegetation.

In this paper, the crop segmentation pipeline developed focuses on identifying the divisions between plots in order to demarcate them, thus avoiding the need for collecting an extensive training dataset to account for changing phenology. Given the divisions between arable crops in experimental settings are almost always straight and most likely in two orientations, a relatively simple technique can be adopted to detect these division lines. Primitives of the division lines can be detected by some form of edge detection such as that of Canny (1986) on the optical imagery or derived DSM. Given that the division lines occur at regular intervals, we hypothesize that edge parameterization may be reducible to a smoothing factor such as the Gaussian envelope used in Canny edge detection. Assuming edge like features are at least partially detected, all that remains is to delineate the complete lines demarcating the divisions, which can be carried out with classical image processing techniques such as the Hough transform or Random Sample Consensus (RANSAC) as used effectively in the close-range industrial approaches of (Bai et al., 2013; Jiang et al., 2016; Vidović et al., 2016). We hypothesize that where segment boundaries require further refinement, level set methods can be used such as those seen in Butenuth and Heipke (2012) and Yan and Roy (2016) can be used to deform segments to more satisfactory boundaries.

## 2. Materials and Methods

### 2.1. Unmanned Aerial System and Flight Planning

**Figure 1:**
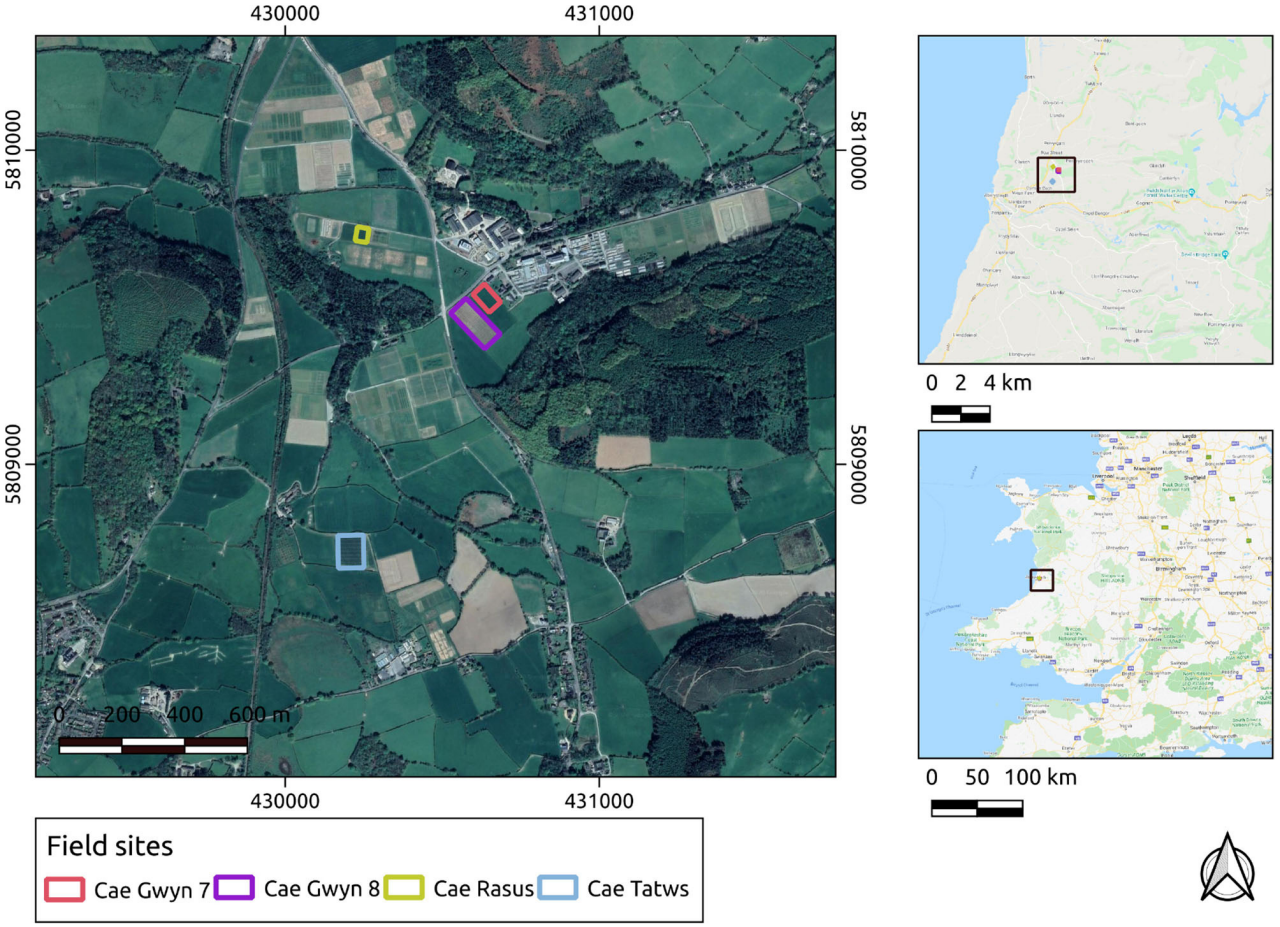
Study sites in their local, regional, and national contexts. Basemap layers are provided by Google.

The UAS-derived datasets were collected over research fields owned by the Institute of Biological, Environmental and Rural Sciences (IBERS, Aberystwyth University), Mid-Wales. The UAS-derived datasets were collected at various points during the growing season of 2018-19 and 2019-20 at midday on each occasion. During the 2018-19 season, the surveys were carried out with a DJI Inspire 1 v2. In the 2019-2020 growing season the surveys were carried out with a DJI M210 v2. In both cases the imagery was captured using a DJI Zenmuse x5 camera payload. The survey flights were planned using Drone Deploy software. The field sites' local, regional and national contexts are displayed in Figure 1.

The site names, conditions, output data and survey characteristics are summarized below in Table 1.

**Table 1:** Study site names, cereal type, phenology, collection date, UAS-derived data type used, and flight height above ground level (AGL).

**Site**	**Cereal**	**Phenology**	**Date**	**Data**	**AGL**	**Overlap**
Cae Tatws	Oat	Senescence	16/07/19	Ortho	50 m	85%
Cae Gwyn 8	Oat	Mid-season/flowering	28/06/18	Ortho	25 m	85%
Cae Gwyn 7	Oat	Emergence	07/02/20	Ortho	40 m	80%
Cae Rasus 4	Oat, Barley,	Emergence	20/01/20	DSM	20 m	80%
	Wheat, Rye					

### 2.2. Photogrammetry

The structure from motion workflows are carried out with the functionality of the MicMac photogrammetry library (Rupnik et al., 2017). The use of open-source methods is of key importance in research for repeatability and the evolution of methods within the field. Parameters were trialed at the bundle adjustment and dense point-cloud stages on a subset of the whole dataset to establish those most suitable for generating outputs for the entire dataset. For further details of the SfM implementation used in this study (see Pierrot Deseilligny and Clery, 2012; Rupnik et al., 2017). The SfM workflow typically consists of four basic stages:

1. Feature extraction and tie-point generation
2. Camera calibration and relative image orientation
3. Bundle adjustment using in flight GPS and/or GCPs
4. Dense point cloud generation
5. Ortho-mosaic generation.

### 2.3. Crop Plot Segmentation

The basis of the image processing pipeline is the Hough transform and line detection, a method developed from the seminal work of Hough (1962) which has been further developed through the years. For a modern review of Hough transform and line detection (see Mukhopadhyay and Chaudhuri, 2015). The Hough transform and line detection are classical image processing techniques which detect straight lines within binary imagery through voting in a parametric space (Mukhopadhyay and Chaudhuri, 2015). The algorithm represents lines in Hesse normal form (1), that is, distance from the image origin and angle coordinate (Mukhopadhyay and Chaudhuri, 2015). A line is expressed in the polar coordinate system as in Equation 1.

(1) $$\begin{array}{r} y=\left(-\frac{\cos\theta }{\sin\theta }\right)x+\left(\frac{r}{\sin\theta }\right) \end{array}$$

Groups of possible lines centered on each pixel appear in the parameter space as sinusoidal form. The intersection points of multiple sinusoidal lines within the parameter space indicate the likelihood of “real” detected lines within the image as these are shared by multiple pixels. The Hough algorithm requires an image of primitive features that at least partially represent the objects of interest. We apply edge detection algorithms to produce the primitives. To mitigate against spurious line detection, the perpendicular lines are detected separately then the results combined. Furthermore, the line detection is constrained to a user defined polygon of the field extent, the orientation of which is detected by non-zero pixels. The orientation of the major and minor axis of the area of interest are used to determine the angles searched by the standard Hough algorithm. The probabilistic variant of the Hough algorithm was not used due to poorer line detection and the prevalence of discontinuous lines. With segments formed by the intersection of Hough-lines, further reduction is carried out using areal parameters. Finally optional boundary refinement can be carried out using level-set methods.

**Figure 2:**
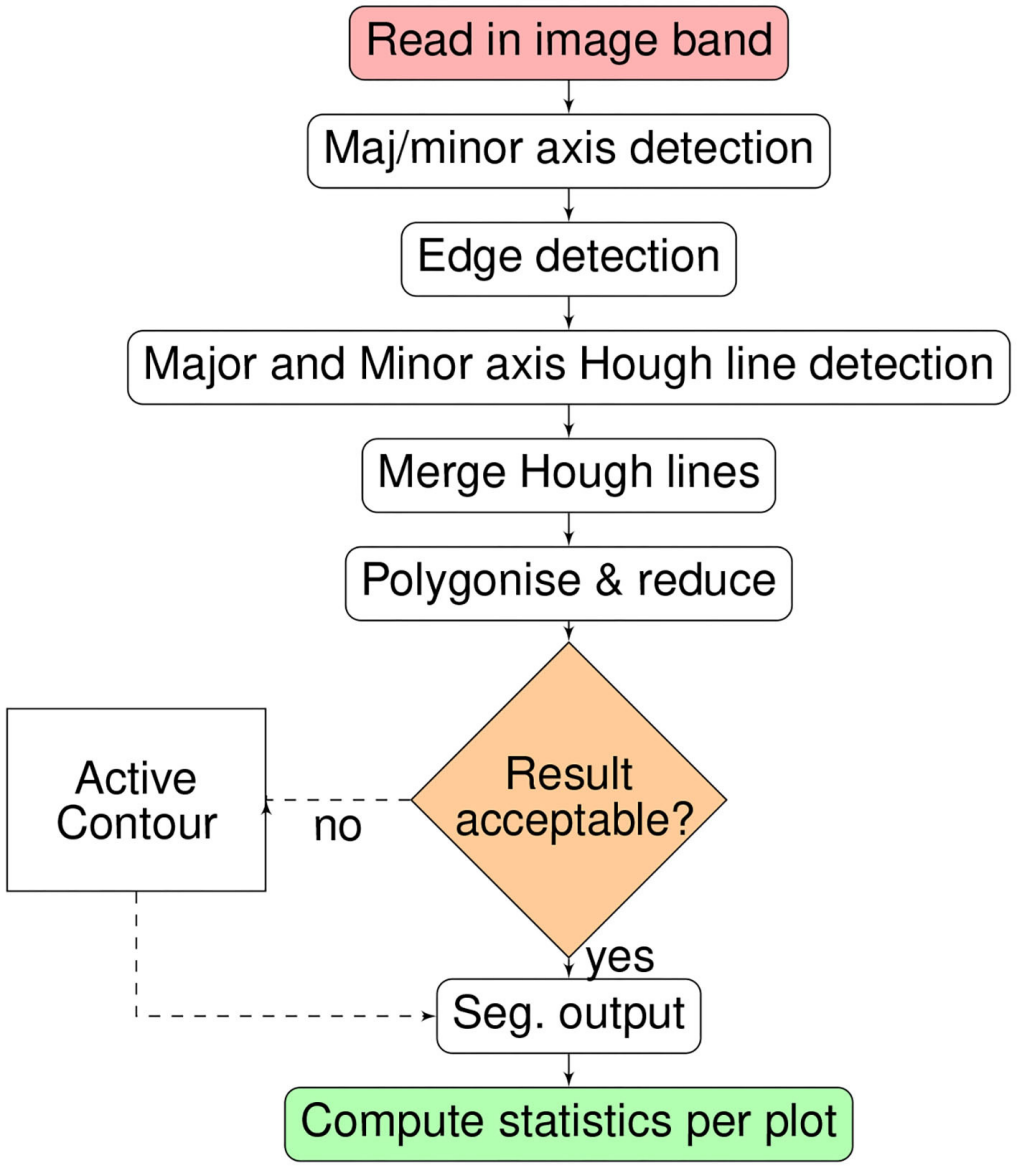
Flow chart summary of the crop segmentation processing chain.

The full processing chain for crop plot segmentation is summarized as a flow chart in Figure 2. The processing-chain is based on the premise of detecting the lines between crop-plots and segmenting the areas within line intersections. Either the optical or derived DSM may be used.

The basic premise of the plot segmentation algorithm is that the crop plots/rows form a grid-like pattern, which can be delineated by the detection of intersecting lines that demarcate the boundaries of each plot. The algorithm requires a cropped image (by GIS polygon) or GIS polygon mask of the field of interest to constrain the line detection. Specifically, the orientation of the major and minor axis of the field are used to constrain the Hough parameter space, limiting line detection to those near-parallel with the major and minor axes. The complete crop plot segmentation function is implemented in python within the Geospatial-learn library (Robb, 2017), itself primarily dependent on the scipy/scikits ecosystem (Virtanen et al., 2020) and GDAL/OGR library (GDAL OGR contributors, 2020).

Binary edge features are produced using either the Canny or Phase Congruency (PC) edge detection algorithms (Canny, 1986; Kovesi, 1999). The Canny-algorithm is based on a combination of spatial filtering and hysteresis thresholding. The density of edges resulting from the Canny algorithm are controlled by the sigma and hysteresis threshold parameters (Canny, 1986). The sigma parameter (σ) dictates the Gaussian envelope used to smooth the input image prior to edge detection (Canny, 1986). Classification of edges is initially based on the sobel approximation of image gradient and classification of pixels based on the gradient orientation. The hysteresis parameters then denote the definitive or upper and connected or lower of gradient intensity that constitute edges (Canny, 1986). In other words, the lower threshold values are only valid if connected to areas of those of the upper/definitive value. Canny (1986) suggests a ratio of 2:1 for the hysteresis parameters, hence we follow this suggestion reducing our parameter set to only σ and the upper threshold, with the lower defined as half of the upper value. The PC algorithm by contrast operates in the frequency domain, and the agreement of phase, detected at multiple scales is used as an edge intensity map (Kovesi, 1999). As with the Canny algorithm, non-maxima suppression and hysteresis thresholding are used to extract a final single pixel width edge map. Whilst slower to process, unlike the Canny algorithm, PC is not susceptible to local contrast variations. Edge detection can also be performed for both major and minor axes of the field separately, as the frequency of useful edges may vary on different axes. This would be applicable where the plot divisions are more frequent along one axis of the field than the other. With both edge approaches we hypothesize that the smoothing (σ) parameter may be sufficient to detect lines from if tuned to the frequency of features within the imagery.

Hough line detection is performed for each edge image with the vertical and horizontal lines written to the image and merged. The underlying implementation of the Hough transform is that of the Scikit-Image library (van der Walt, 2014). The lines are converted to polygons/segments, defined by their enclosed areas to produce an initial segmentation. The grid of polygons is reduced to only the crop plots of interest with a minimum/maximum area parameter, where all polygons that do not fit this criteria are discarded. If required, closer adherence to the crop edges may be obtained using active contours where each segment is deformed according to the minimization of an energy defined by the pixel values “acting upon” the contour border from outside and with the segment as well as along it's boundary (Chan and Vese, 2001). The use of active contours has been demonstrated by Yan and Roy (2016) in the refinement of field boundaries from manually digitized data and by Butenuth and Heipke (2012) in the refinement of preliminary segmentation results. The implementation used in this study is the image morphology derived version, which is more computationally efficient than the original partial differential equation solution as developed and implemented by Marquez-Neila et al. (2014).

Initial experimentation was performed on a subset of the Cae Tatws site data to establish parameter combinations would result in successful delineation on a relatively small scale. The processing was then expanded to the entire field within which the initial subset was contained. The initial dataset had reasonably well-defined boundaries between plots and were aligned close to the axes of the image. Hence, it was important to test the method on more poorly defined plots and challenging conditions. The next test images were therefore less well-defined and aligned at angles far from the image axes at various stages of growth.

### 2.4. Accuracy Assessment

Segmentation accuracy was measured using the F-1 score, which is the harmonic mean of precision and recall, where tp are true positives, fp are false positives and fn are false negatives and β is the weight assigned to precision and recall. For this study, β is left at 0.5 giving equal weight to precision and recall in the F-1 score.

(2) $$\begin{array}{r} \text{precision}=\frac{tp}{tp+fp} \end{array}$$

(3) $$\begin{array}{r} \text{recall}=\frac{tp}{tp+fn} \end{array}$$

(4) $$\begin{array}{r} F_{\beta }=\left(1+\beta^{2}\right)\frac{\text{precision}\times \text{recall}}{\beta^{2}\text{precision}+\text{recall}}. \end{array}$$

The validation layers are complete manually digitized maps from the UAS imagery, as no finer resolution imagery is available. The datasets and their corresponding number of digitized crop plots are summarized in Table 2.

**Table 2:** Datasets with the number of corresponding crop plots digitized for each validation set.

**Site**	**Number of digitized crop plots**
Cae Tatws	645
Cae Gwyn 8	989
Cae Gwyn 7	180
Cae Rasus 4	84

## 3. Results

### 3.1. SfM

The relative orientation and GPS-aided bundle adjustment were carried out using different lens distortion models to ascertain the model type that minimized the re-projection error. For each imagery set, the Fraser lens model (Fraser, 1997) consistently produced the lowest pixel residual. The results of the bundle adjustment are displayed in the Table 3.

**Table 3:** Bundle adjustment RMSE for each dataset (in pixels).

**Site**	**Residual in pixels of GPS-aided bundle adjustment**
Cae Tatws	0.70
Cae Gwyn 8	0.78
Cae Gwyn 7	0.82
Cae Rasus 4	0.89

### 3.2. Line Detection and Plot Segmentation

#### 3.2.1. Experimental Results

**Figure 3:**
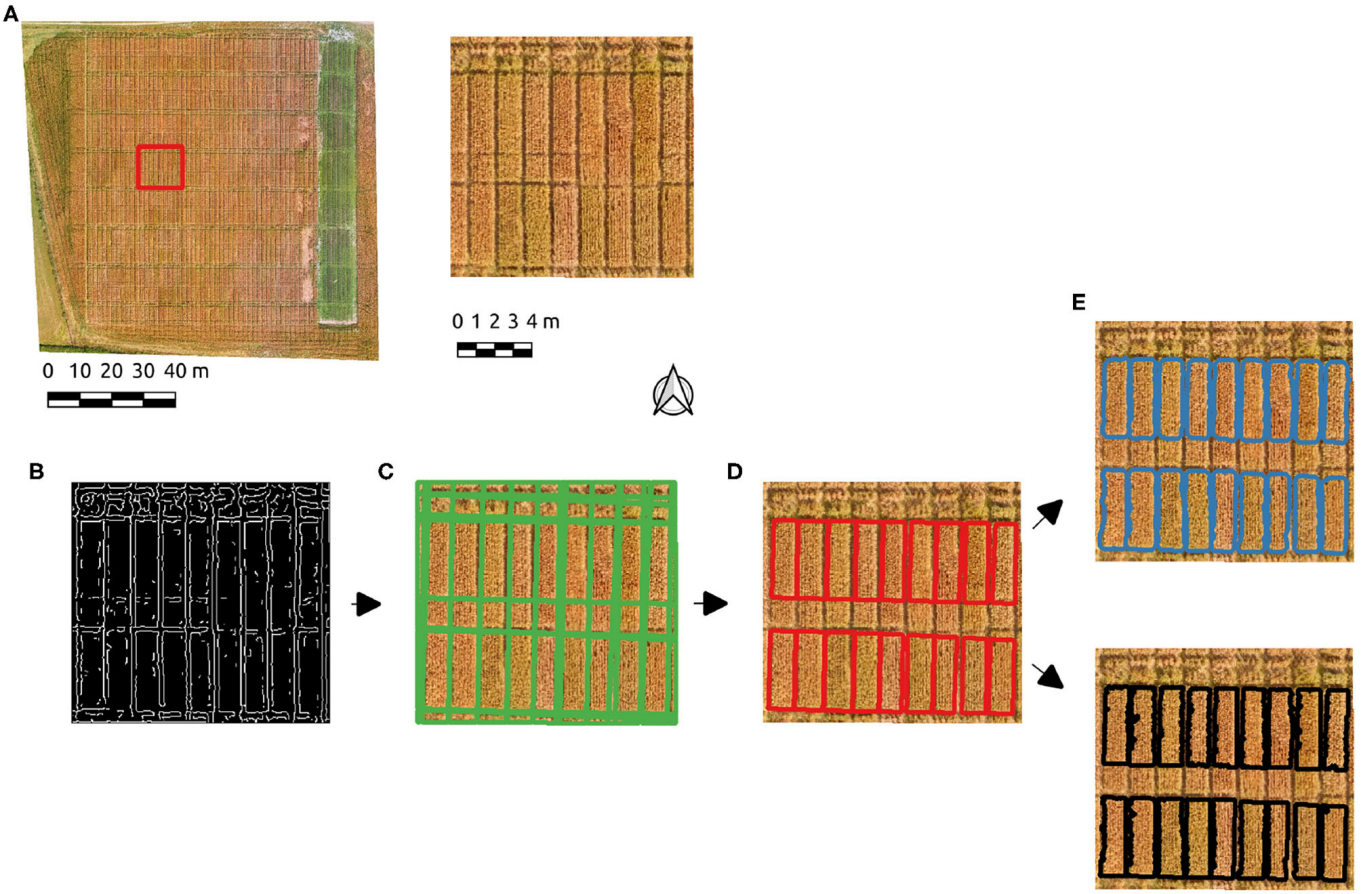
The experimental workflow results; **(A)** the test image subset used, **(B)** Canny edge detection, **(C)** Hough line detection results, **(D)** polygon elimination, **(E)** active contour (blue) and Otsu-based (black) boundary refinement results.

An subset of the Cae Tatws site data was used to trial the algorithm which is summarized in Figure 3, consisting of the test image subset used (Figure 3A), Figure 3B Canny edge image, Figure 3C Hough line detection results, Figure 3D polygon elimination based on area and refinement (Figure 3E) via either active contour and Otsu-based threshold.

After initial experimentation, Canny edge parameters of σ = 2 and hysteresis threshold of 50 produced edges (Figure 3B) resulting in a successful Hough line detection for the test image, where all relevant boundaries were well-approximated (Figure 3C). A single parameter σ = 4 also produced the correct number of plots, but more poorly aligned boundaries. PC-based parameters produced well-defined boundaries with the parameter σ = 2. The Hough line detection provided an initial delineation of the crop segments, which included ancillary segments present as a result of the planting and division patterns. Each crop plot of interest is of approximately 3.5 m2, hence a minimum area of segment was set to eliminate those below this threshold (Figure 3D). The boundaries approximate the plots, but tend to over-segmentation due to the incidence of light on the plots in the imagery. Given this occurs over areas of shadow and/or soil, a foreground/background segmentation was tested to enhance the initial Hough-based boundaries. Boundary refinement was tested via both active contours and Otsu's threshold method on the Canny-derived polygons, but neither resulted in a consistent overall improvement over initial results (Figure 3E). By using PC edge detection the effect of local contrast is removed and the shift in boundaries is eliminated.

#### 3.2.2. Scaled-Up Results

Using the same basic workflow, crop plots were segmented from the field-scale datasets. Table 4 lists algorithm parameters for the best performing segmentation on each dataset.

**Table 4:** Parameter sets for crop plot, where; CN, Canny; PC, Phase-Congruency; ma, minimum area in square meters, hysteresis threshold; Mj,Mn, Major and Minor-Axis.

**Dataset**	**Edge algorithm**	**σ**	**Thresh**	**ma**
Cae Tatws	CN	4		3.5 m2
Cae Tatws	PC		1	3.5 m2
Cae Gwyn 8	CN	6		2.4 m2
Cae Gwyn 7 Mj	CN	4	6	
Cae Gwyn 7 Mn	CN	40		8 m2
Cae Rasus 4	CN	10		10 m2

The F-1-based accuracy metrics for each field site are summarized in Table 5. The following section will interpret each field site in turn by name from top to bottom of Table 5.

**Table 5:** Accuracy scores using the F1 metric for each of the fields.

**Dataset**	**Edge algorithm**	**Precision**	**Recall**	**F1-score**
Cae Tatws	CN	88	92	90
Cae Tatws	PC	91	97	94
Cae Gwyn 8	CN	88	91	89
Cae Gwyn 7	CN	96	83	89
Cae Rasus 4	CN	98	81	89

The entire Cae Tatws site was used as the first field-wide test. To achieve complete coverage of every plot required only a slightly different Canny parameter set than that of the experimentation subset, which was; σ = 4, and min-area = 3.5 m2. Crop plot segmentation accuracy scores were 0.88 (precision), 0.92 (recall), 0.9 (F1) for the Canny-based pipeline.

**Figure 4:**
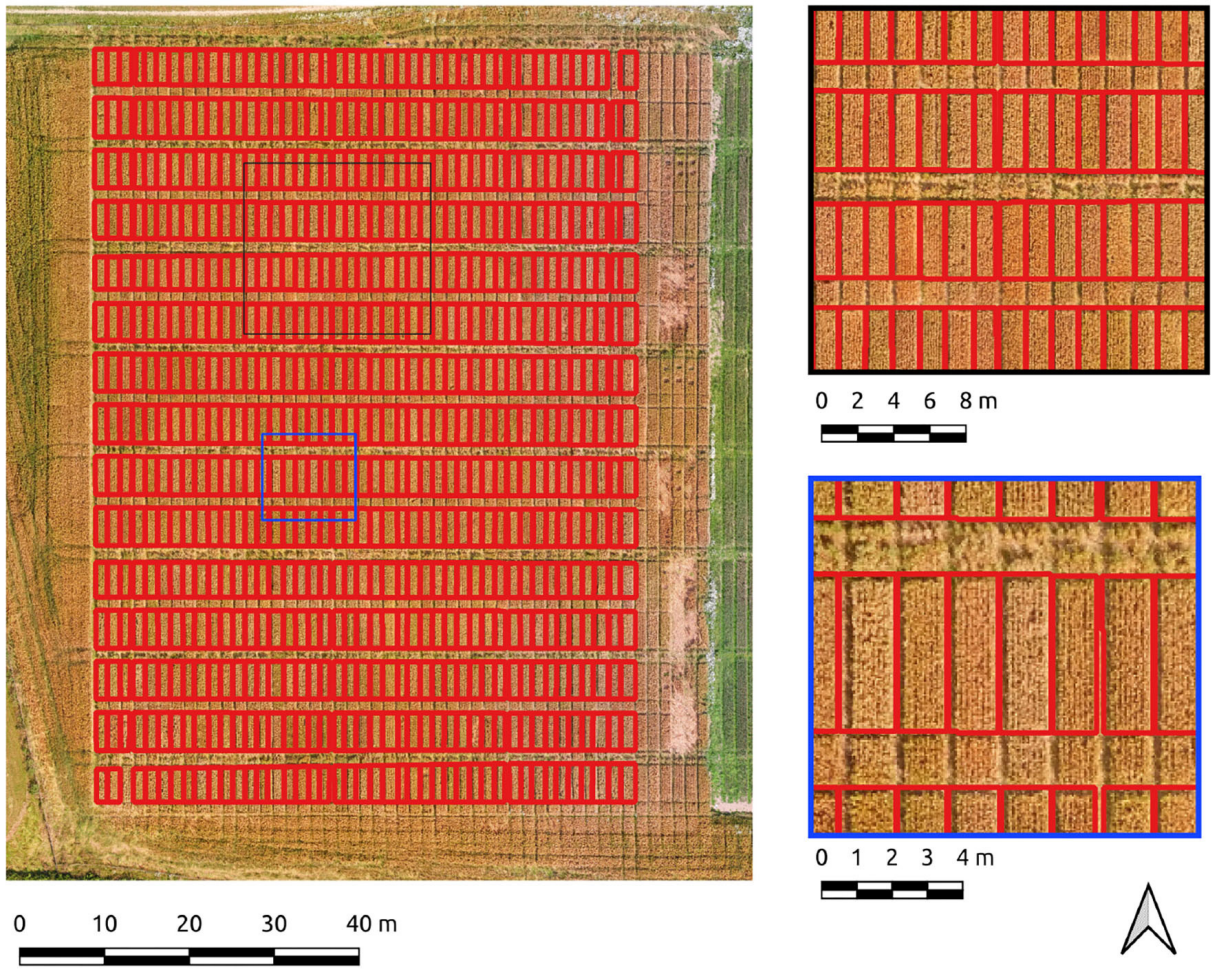
Segmentation results on the Cae Tatws site dataset using Canny edge detection.

An overall F1-score of 0.9 indicates generally well-defined crop plot segments. Visual inspection reflects this, with a drift is evident in areas of shadow due to local contrast variations (Figure 4). Hence, there is a tendency for the segment edges to delineate the outer edge of the plot shadow. This drift is reflected numerically in a higher rate of commission error (0.22) or inversely, a lower precision score (0.88) for crop plot segments.

**Figure 5:**
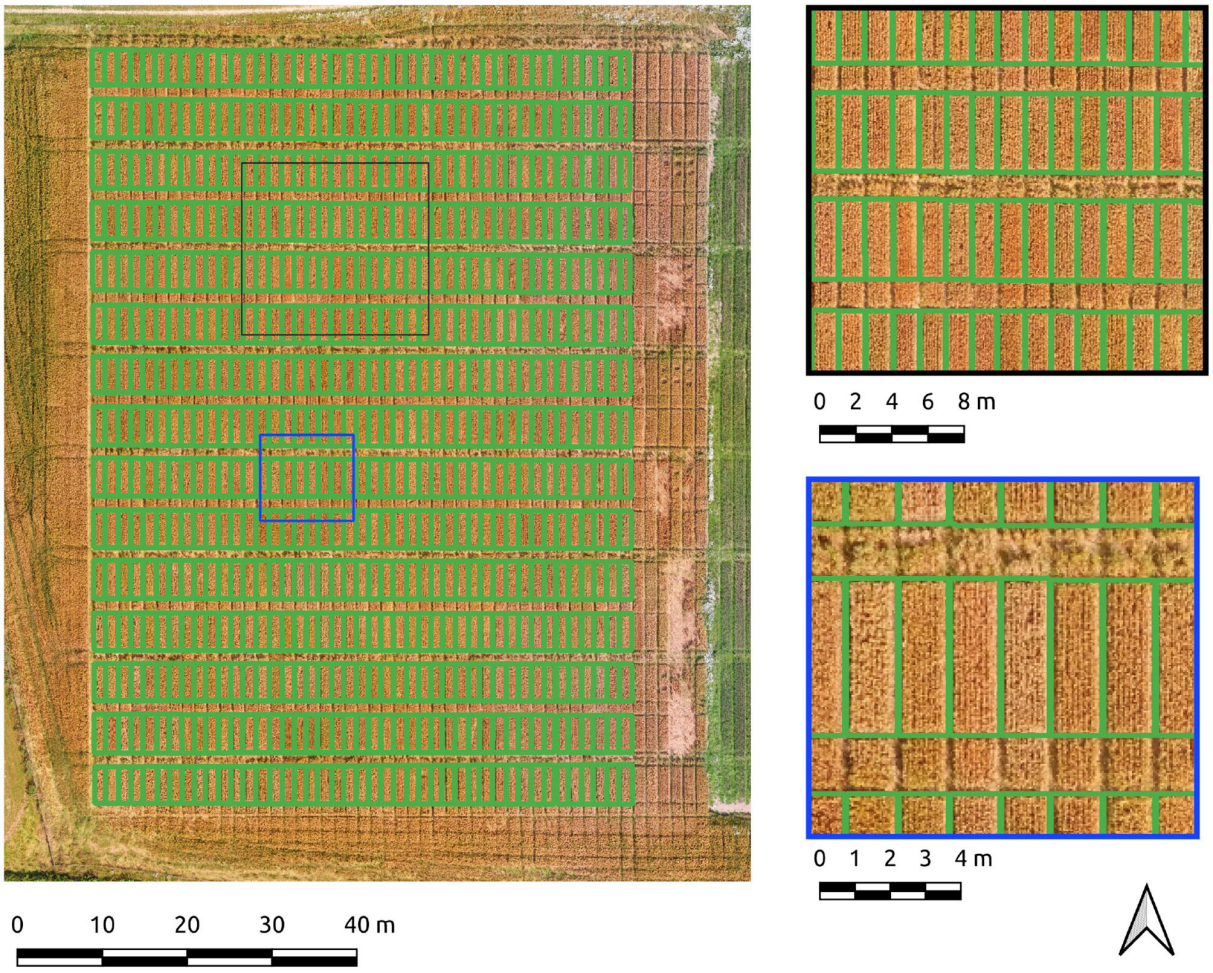
Segmentation results on the Cae Tatws site dataset use PC edge detection.

The same data was segmented using the PC edge detection with improved results, as shown by an overall F1-score of 0.91. Segment edges are well-defined and occupy a more central position between the plots than the Canny approach (Figure 5). The improved spacing is the likely explanation for the marked improvement in precision (0.91) and recall scores (0.97). Of particular note is the 0.97 recall score, evidence that the majority of every plot pixel has been captured by the segmentation. Precision is still high, indicating a commission error of only 0.09 for crop plot segments.

**Figure 6:**
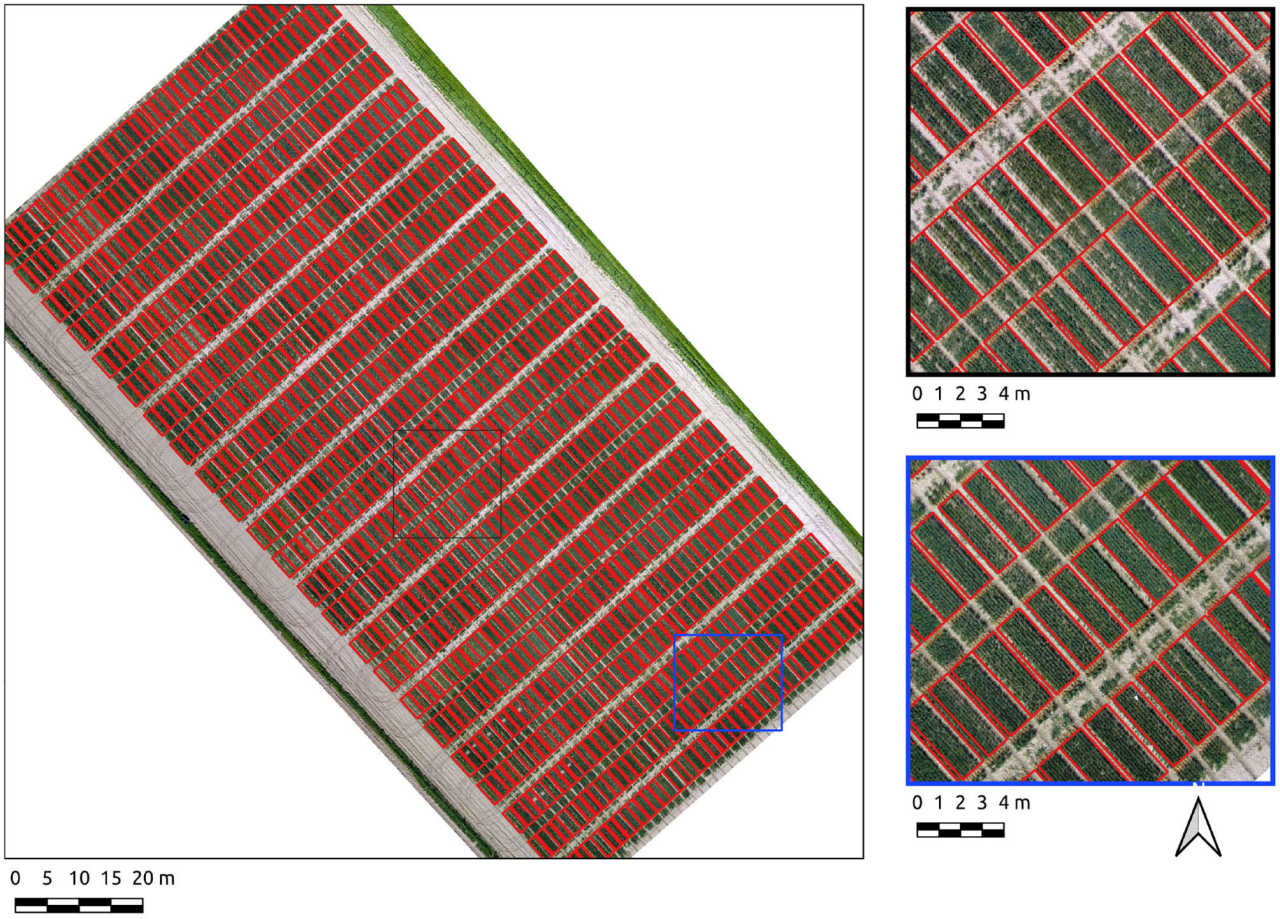
Segmentation results on the Cae Gwyn 8 site crop plots set using the Canny-based approach.

Results for the Cae Gwyn 8 site, a mid season flowering-stage field are now presented. The Canny-based approach was more effective in this context with PC-based edge detection not yielding usable results. The Canny-based pipeline was used with an overall F1-score of 0.89. The parameter set was σ = 6 and min-area = 2.4. The recall score is high (0.91) showing the majority of plot pixels are covered. The precision score (0.88), whilst high, is indicative of over-segmentation in some areas along the vertical axes of the plots (Figure 6). These errors of commission are attributable to plot division areas where the side of plots have been missed during the line detection stage (Figure 6, insets).

**Figure 7:**
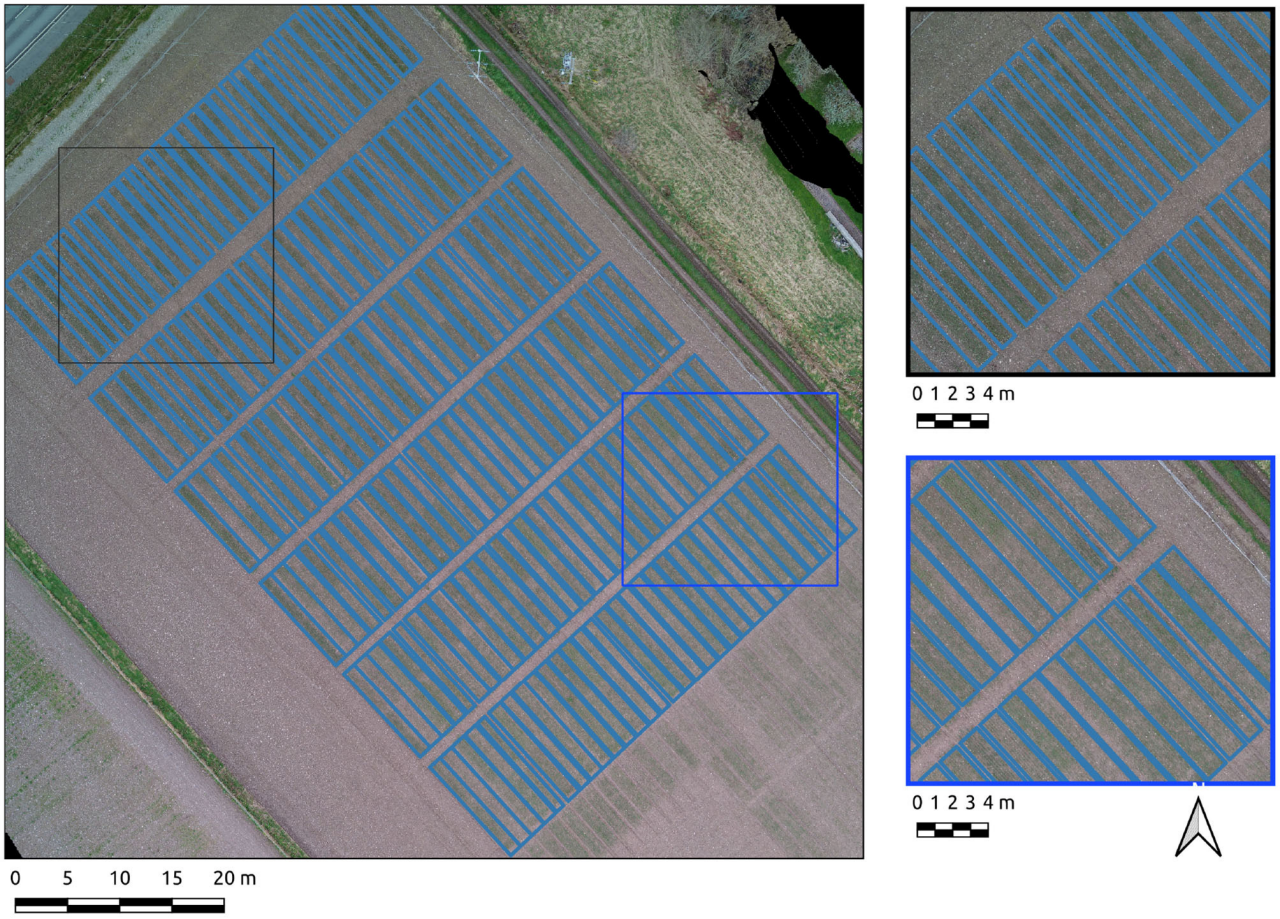
Segmentation results on the Cae Gwn 7 site plots set using the Canny-based approach.

Results from the Cae Gwyn 7 site, an emergence-stage field are now presented. The Canny-based approach was more effective in this context with PC-based edge detection not yielding usable results. Separate parameter sets for each axis were required due to both noise and differing frequencies of plot boundaries per axis. As was experienced with the previous datasets, simply using the σ parameter almost yielded a complete segmentation (σ = 4, σ = 40, respectively) save for one division along the major axis for the field. Consequently, a hysteresis threshold of 6 was required for complete segmentation along the major axis. An overall F1-score of 0.89 indicates good generalized segmentation performance. However, there is a greater difference between precision (0.96) and recall (0.83) for crop plot segments which indicates a greater rate of omission error (0.16) compared to a commission error of 0.05. The combination of higher omission error and low commission error indicates under-segmentation, which is reflected by visual inspection where plots are under-segmented along their major axis (Figure 7).

**Figure 8:**
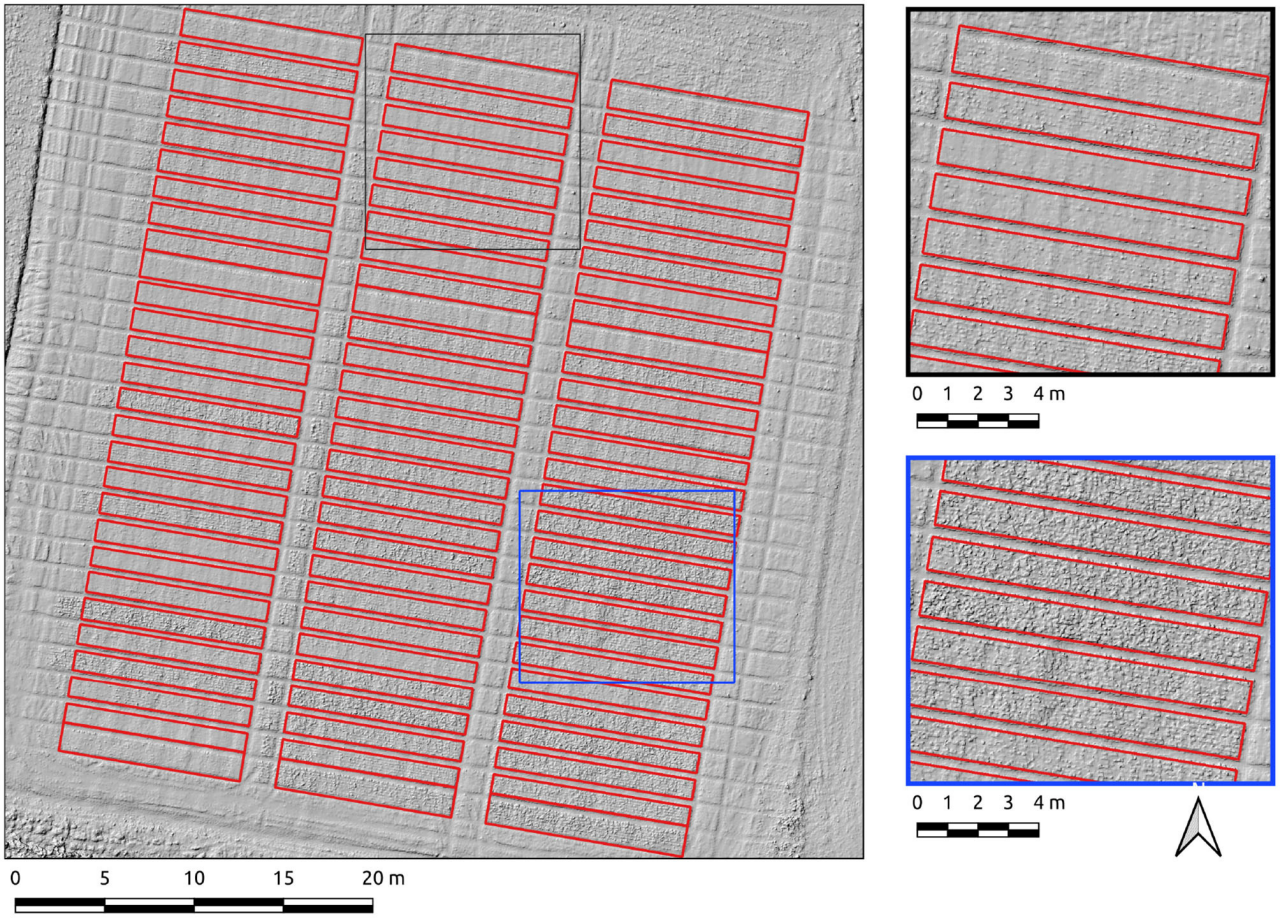
Segmentation results on the Cae Rasus 4 site crop plots set using the Canny-based approach.

Results for the Cae Rasus 4 site, an emergence-stage field, are now presented where the data used is an SfM-derived DSM. The Canny-based approach was more effective in this context with PC-based edge detection not yielding usable results. The Canny-based pipeline was used with an overall F1-scores of 0.89. The parameter set was σ = 10 and min-area = 10. The precision score (0.98) indicates relatively few errors of commission. The recall score (0.81) is indicative of under-segmentation in some cases (Figure 8). These errors of omission are attributable to edge detection demarcating the upper break of slope on each of the plots (Figure 8, insets).

## 4. Discussion

The accurate segmentation of crop plots is important to localize the spectral and spatial characteristics that indicate phenological state for particular genotypes or treatments. Within each plot segment, the magnitude of radiometric values, canopy height, pixel texture and indeed the presence or absence of plants from areas within the plot give a proxy indication of the efficacy of the treatment regime or crop variety in question. The methods presented in this study integrated edge detection and Hough line detection to segment crop plots. The approach has been shown to consistently produce plot segmentation accuracy of over 89%. This high level of accuracy gives confidence for its use in extracting key plot parameters for use in crop monitoring.

Accurate crop plot segmentation in this study is reliant on a representative edge detection to ensure enough lines are detected via Hough transform that constitute segments. The Canny edge detection algorithm proved to be the most flexible, the PC approach also had merit. From the evidence in this study, the PC algorithm results in a better line detection provided the underlying imagery has relatively clear boundaries and adheres better to the center of the crop divisions, due to less susceptibility to local contrast. Performance is inferior to the Canny-based approach where image noise is more evenly distributed. On balance therefore, we would recommend the use of the Canny-based approach in most situations. It was hypothesized that the σ parameter may have been sufficient to detect edges appropriate for line detection as with a greater Gaussian envelope, high frequency features are reduced. The increase of σ = 2−4 from the test subset to the entire field at Cae Tatws is likely due to a greater range of pixel values in the larger dataset requiring a greater Gaussian envelope. The sole use of the sigma parameter largely held true for the 4 field-scale datasets, with only a minor adjustment required on Cae Gwyn 8, suggesting in most cases parameterization is relatively simple. The detected edges need not be comprehensive as the resulting Hough lines intersect the entire image. This has the advantage of being deployable when only plough lines or planting rows are partially visible, which was the case in all three datasets, whereas Ahmed et al., 2019; Khan and Miklavcic, 2019; Tresch et al., 2019; Matias et al., 2020 rely upon the presence of well-spaced plants. Indeed, Ahmed et al. (2019) assume the crop plot segments identified are discrete, which is unlikely to be effective when some adjacent plots canopies coalesce.

Our approach only requires limited parameterization (σ, minimum area), whereas the most closely related studies (Khan and Miklavcic, 2019; Tresch et al., 2019; Matias et al., 2020) all require a parameter-based manual construction of the entire crop plot grid, involving the number of columns, rows, their dimensions, positioning and orientation. This makes any comparison difficult, as these studies are manual constructions, with no plot detection made from the image values. Whilst the parameter-based manual creation of a crop plot grid from scratch represents a minor, albeit helpful improvement over manual digitizing, it could be achieved via standard GIS methods. Khan and Miklavcic (2019) enhance their grid construction tools with the facility to fine tune each plot position via energy minimization, but apply this to synthetic crop plot displacements which may not be representative of applied scenarios. The method developed in this paper removes the need for multi-stage manual construction of plot grids as utilized in recent studies, only requiring the tuning of the edge parameter. Most importantly, we applied our method to multiple examples of UAS-derived imagery captured over a variety background cover and emergent conditions, ensuring the wider applicability of this work. Additionally we show that the method can be applied to both optical and DSM data.

Useful future development of this work concerns both the existing parameters and integration with other platforms. The estimation of the σ parameter from the edge detection stage of this work would, in most instances, remove the need for parameterization and thus ease the use of our algorithm. Segmentation of the field interest would also enhance this study, which could possibly be achieved via the integration of satellite born data or even from the UAS data itself. Furthermore, the expansion of this work to fine spatial-resolution satellite data may prove to be useful provided the crop plots are discernible at the resolution of current sensors.

This paper provides a consistently high performing approach for delineating cereal crop plots with minimal input from the user, representing a significant advance over largely manual previous attempts.

## 5. Conclusions

Improving techniques in precision agriculture is integral to ensuring global food security. UAS are playing an increasingly prominent management and monitoring role in precision agriculture due to their affordability and efficiency over traditional field-base monitoring. Central to providing valuable information from UAS in agricultural practices is the user's ability to define discrete crop units. This paper provides for the first time, a consistently high performing approach for delineating crop plots with minimal input from the user. In this study, we propose a crop plot segmentation pipeline consisting of edge detection, Hough line detection and segment reduction. We tested our pipeline in a variety of circumstances to ensure wide applicability, with heterogeneous backgrounds, growth stages, crops, and image quality. Our results show segmentation accuracy of over 89% are regularly achievable with minimal parameterization. The segmentation of crop plots from UAS-derived imagery forms an important of management and monitoring in experimental agriculture, and our method could be readily deployable over extensive areas in this context.

## Data Availability Statement

The raw data supporting the conclusions of this article will be made available by the authors, without undue reservation.

## Author Contributions

CR wrote the article, conceived and designed the algorithmic solution, and implementation of software. AH and JD research the discussions and article internal review and additions. JB UAS pilot and technician, planned and carried out all UAS surveys. All authors contributed to the article and approved the submitted version.

## Conflict of Interest

The authors declare that the research was conducted in the absence of any commercial or financial relationships that could be construed as a potential conflict of interest.
